# Seroprevalence of diphtheria toxoid IgG antibodies in children, adolescents and adults in Poland

**DOI:** 10.1186/1471-2334-13-551

**Published:** 2013-11-19

**Authors:** Aleksandra A Zasada, Waldemar Rastawicki, Natalia Rokosz, Marek Jagielski

**Affiliations:** 1 Department of Bacteriology, National Institute of Public Health – National Institute of Hygiene, Chocimska 24, Warsaw 00-791, Poland

**Keywords:** Diphtheria, IgG antibodies, Diphtheria toxin, Vaccination

## Abstract

**Background:**

Recommendations for diphtheria immunization are to apply an effective primary immunization in infancy and to maintain immunity throughout life. Immunity against diphtheria depends primarily on antibody to the diphtheria toxin. This study evaluated the seroprevalence of IgG diphtheria antitoxin in sera of healthy children, adolescents and adults in Poland.

**Methods:**

A total of 1387 serum samples collected between 2010 and 2012 from individuals with ages ranging from 1 month to 85 years were investigated. Antibody concentrations were measured with an enzyme-linked immunosorbent assay (Anti-Diphtheria Toxoid ELISA IgG, Euroimmun, Germany).

**Results:**

The results showed that among 1387 individuals examined, 547 (39.4%) had anti-diphtheria toxoid IgG antibody levels below 0.1 IU/ml (36.9% ≤18 years and 40.5% >18 years old, respectively). The 212 (50.8%) children and 542 (55.9%) adults showed only basic protection (0.1-1.0 IU/ml) and need immediate booster. High levels of anti-diphtheria toxoid IgG antibodies (>1.0 IU/ml) were found more often in children and adolescent (12.2%) than in adults (3.6%) and this was statistically significant (P < 0.05). The proportion of seronegatives (< 0.1 IU/ml) in children below 2 years old, adolescents and young adults to 25 years old decreased from 53.5% to 17.4%. However, in older individuals the seronegative proportion tended to increase with age, from 22.7% in adults (26–30 years old) to 67.1% in subjects > 60 years old. Characteristically, in individuals > 40 years old high levels of anti-diphtheria toxoid IgG antibodies (>1.0 IU/ml) were not seen. There were no statistically significant differences in results in relation to gender.

**Conclusions:**

The present study showed inadequate immunity levels to diphtheria amongst the Polish population, especially in adults > 40 years old and children ≤ 2 years old. To prevent reemergence of diphtheria an information campaign reminding people about recommendations concerning diphtheria booster vaccination in adults should be conducted. Moreover, the immunogenicity of the DTP vaccine used in Poland should be verified.

## Background

Diphtheria is a severe and potentially fatal disease caused by toxin-producing strains of *Corynebacterium diphtheriae*, *Corynebacterium ulcerans* and *Corynebacterium pseudotuberculosis*. Before the introduction of active vaccination in 1940s, diphtheria was endemic in most European countries [1]. Currently, the disease appears to be well controlled in developed countries but is still endemic in Africa, Asia and Eastern Europe [2]. Humoral immunity against diphtheria depends primarily on formation of specific IgG antibodies to diphtheria toxin, which may be induced by natural infection, passive or active immunization. As diphtheria has become rarer, opportunities for acquisition or reinforcing natural immunity have also been reduced [3,4]. In most European countries diphtheria vaccine is included in the recommended vaccination schedule. In Poland the diphtheria vaccination schedule comprises 7 doses administered at 2 months, 3–4 months, 5–6 months, 16–18 months and then 6, 14 and 19 years. According to World Health Organization data, more than 95% of children are fully vaccinated against the disease in Poland. However, the level of antibodies decreases with time and adults may again become susceptible to diphtheria due to reduced opportunities for boosting through subclinical infections. A large pool of susceptible persons creates an epidemic potential, as demonstrated by the last diphtheria epidemic that occurred in the early 1990s in the countries of the former Soviet Union when more than 50 000 cases were recorded at the peak of the epidemic. During this epidemic adolescents and adults were mainly affected, most of whom would have been previously vaccinated [4-7]. Moreover, during the last decade the number of diphtheria cases due to *C. ulcerans* has increased in Europe. For example, 63% of toxigenic corynebacteria isolated in France in 2002–2008 and in United Kingdom in 2000–2009 were *C. ulcerans*. The reservoir hosts of this species are domestic cats and dogs [8,9].

In Poland the last diphtheria case was recorded in 2000 and the previous 9 cases were recorded in 1996 [10]. In the present study we determined the immune status against diphtheria in different age groups of the population after a period of over 10 years with no cases of diphtheria in Poland.

## Methods

### Study population

A total of 1387 serum samples were collected to examine the specific anti-diphtheria toxoid antibody levels. Written informed consent was obtained from participants, parents or guardians. The serum bank comprised samples collected between 2010 and 2012, from individuals living in different regions of Poland aged from 1 month to 85 years (median, 26 years). Samples from the group aged 0–18 years (n = 417) were residual sera from diagnostic laboratories, whereas samples from the adult population (n = 970) included residual sera from diagnostic laboratories (n = 260) and additionally from routine screening tests of healthy blood donors (n = 390), forest workers (n = 122) and pregnant women (n = 198). Diphtheria vaccination history of the tested individuals was not available. Data on gender were available from 1047 individuals (544 females and 503 males). Precise data on age were not obtained from forest workers and most of the blood donors.

### Determination of diphtheria toxoid antibody levels

Diphtheria toxoid IgG-specific antibody levels were determined using a commercial ELISA Anti-Diphtheria Toxoid ELISA IgG (Euroimmun, Germany) selected in previous studies as the most reliable of those anti-diphtheria IgG assays tested [11]. For quantitative evaluation four ready-to-use calibrators - Calibrator 1 (2 IU/ml), Calibrator 2 (1 IU/ml), Calibrator 3 (0.1 IU/ml), Calibrator 4 (0.01 IU/ml) and two control sera (one positive and one negative) were provided in the kit.

The concentrations of the of anti-diphtheria toxoid antibodies in serum samples were determined using a standard curve. For the calculation of the standard curve the OD (optical density) of each Calibrator (y-axis, linear) was plotted against the concentration (x-axis, logarithmic) using Excel (Microsoft). The four Calibrators were calibrated in IU/ml against the International Standard for Diphtheria Antitoxin NIBSC 00/496. The initial dilution of test sera was 1:101. Samples which showed concentrations above the highest standard were further diluted. Results of samples with higher predilution were multiplied by the dilution factor. Manufacturer recommended division of the results into five groups: <0.1 IU/ml (indicating immediate basic immunisation), 0.1-1.0 IU/ml (immediate booster), > 1.0-1.5 IU/ml (booster after 5 years), > 1.5-2.0 IU/ml (booster after 7 years) and > 2.0 IU/ml (booster after 10 years).

### Statistical analysis

The study population was divided into ten age groups: 0–2, 3–5, 6–13, 14–18, 19–25, 26–30, 31–40, 41–50, 51–60 and > 60 years. The arithmetic mean titres, standard deviations and geometric mean titres were calculated using Excel. The statistical significance of the differences was analyzed by Fisher’s exact probability test with Yates’ correction when at least one of the calculated figures was <5. A *P*-value < 0.05 was considered significant.

The studies were approved by Bioethics Committee of National Institute of Public Health – National Institute of Hygiene (reference number 2/2013).

## Results

The distribution of antibodies, arithmetic and geometric mean titres and other statistical parameters in children and adults are presented in Table 1. Among 1387 individuals examined, 547 (39.4%) had levels of anti-diphtheria toxoid IgG antibodies below 0.1 IU/ml (36.9% ≤ 18 years and 40.5% >18 years old, respectively). The 212 (50.8%) children and 542 (55.9%) adults showed only basic protection (0.1-1.0 IU/ml) and require immediate immunisation. In general, the difference in the number of seronegatives and low positives (0.1-1.0 IU/ml) between individuals ≤ 18 years old and adults was not statistically significant (P > 0.05). However, high levels of anti-diphteria toxoid IgG antibodies (>1.0 IU/ml) were more often found in children and adolescent (12.2%) than in adults (3.6%) (P < 0.05) and this was statistically significant. The geometric mean titre (GMT) was low, both in children (0.141 IU/ml) and in adults (0.102 IU/ml). There was no statistically significant difference in diphtheria antibody levels between males and females (P > 0.05).

**Table 1:** Distribution of seroprotection against diphtheria in the Polish population according to age group

**Age groups**	**≤18 years**	**>18 years**	**Total**
Number of persons	417	970	1387
Titre < 0.1 IU/ml	154 (36.9%)	393 (40.5%)	547 (39.4%)
Titre 0.1-1.0 IU/ml	212 (50.8%)	542 (55.9%)	754 (54.4%)
Titre >1-1.5 IU/ml	30 (7.2%)	26 (2.7%)	56 (4.0%)
Titre >1.5-2 IU/ml	18 (4.3%)	6 (0.6%)	24 (1.7%)
Titre > 2.0 IU/ml	3 (0.7%)	3 (0.3%)	6 (0.4%)
Arithmetic mean titre (IU/ml)	0.409	0.274	0.310
Standard deviation (IU/ml)	0.559	0.324	0.414
Geometric mean titre (IU/ml)	0.141	0.102	0.108
Minimum (IU/ml)	0.001	0.001	0.001
Maximum (IU/ml)	4.480	1.740	4.480
Median (IU/ml)	0.210	0.150	0.160

Data presented in the Table 2 show in more detail the distribution of anti-diphtheria toxoid IgG titres in healthy individuals from different age groups. The proportion of seronegatives (< 0.1 IU/ml) in children, adolescents and young adults up to 25 years old appears to decrease from 53.5% to 17.4%. However, in older individuals the seronegative proportion tended to increase with age, from 22.7% in adults aged 26–30 years to 67.1% in subjects > 60 years old. Characteristically, in individuals > 40 years old high levels of anti-diphteria toxoid IgG antibodies (>1.0 IU/ml) were not seen.

**Table 2:** Euroimmun assay results of 965 tested subjects according to age group

**Age groups (years)**	**Number of tested sera**	**Number (percentage) of sera with level of anti-diphtheria antibodies:**				
		**<0.1 IU/ml**	**0.1-1.0 IU/ml**	**>1-1.5 IU/ml**	**>1.5-2.0 IU/ml**	**>2.0 IU/ml**
0-2	114	61 (53.5)	38 (33.0)	10 (8.8)	4 (3.5)	1 (0.8)
3-5	100	36 (36.0	49 (49.0)	7 (7.0)	7 (7.0)	1 (1.0)
6-13	133	41 (30.8)	77 (57.9)	9 (6.8)	5 (3.8)	1 (0,8)
14-18	70	16 (22.9)	48 (68.6)	4 (5.7)	2 (2.9)	-
19-25	69	12 (17.4)	52 (75.4)	5 (7.2)	-	-
26-30	110	25 (22.7)	80 (72.7)	3 (2.7)	2 (1.8)	-
31-40	145	34 (23.4)	103 (71.0)	6 (4.1)	1 (0.7)	1 (0.7)
41-50	80	51 (63.8)	29 (36.2)	-	-	-
51-60	70	46 (65.7)	24 (34.3)	-	-	-
>60	73	49 (67.1)	24 (32.9)	-	-	-

**Figure 1:**
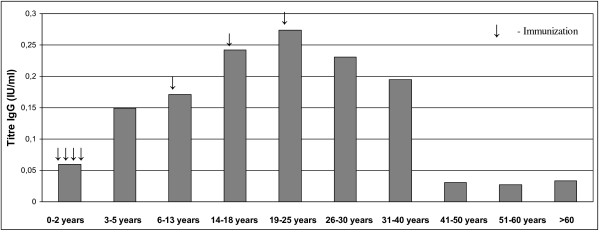
Geometric mean concentration of diphtheria toxoid antibodies in the Polish population according to the age groups.

**Figure 2:**
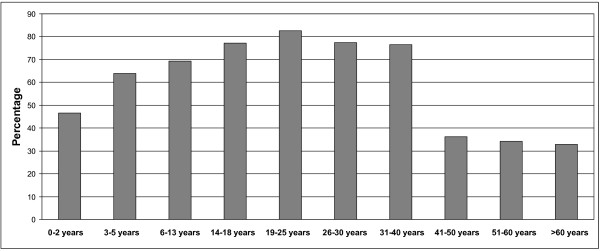
Percentage of subjects with diphtheria toxoid antibody levels ≥0.1 IU/ml in the Polish population according to the age groups.

A similar picture is seen in Figures 1 and 2, which show the geometric mean concentration of anti-diphtheria toxoid IgG antibodies and percentage of subjects with anti-diphtheria toxoid antibody levels ≥ 0.1 IU/ml according to age groups, respectively. The effects of the each booster dose are clearly visible, causing the maximum protection level in individuals aged 19–25 years (GMT = 0.274 IU/ml). In older age groups we can see the decrease of GMT values as well as decrease of the percentage of subjects with protective levels of anti-diphtheria toxoid IgG antibodies. The most notable observation is the dramatic decrease of protection in individuals > 40 years old.

## Discussion

Although diphtheria has occurred sporadically in Western Europe, the risk of importation of the disease from endemic regions is increasing together with the increase in the risk of infection via mass tourism to diphtheria endemic countries. Moreover, domestic animals, such as cats and dogs, were identified as a new source of human infections of diphtheria toxin producing *C. ulcerans*[1,8]. For these reasons a protective level of diphtheria toxin antibodies should be maintained in populations.

In this study the overall proportion of susceptible persons was 39.4%. This is of great concern as such a high percentage without a protective level of anti-diphtheria antibodies creates an epidemic potential. WHO outlined that to achieve sufficient herd immunity a minimum immunity rate of 90% in children and 75% in adults is required [12]. The DTP (the diphtheria-tetanus-pertussis vaccine) vaccination coverage in children in Poland has been 99% since 2002, and in previous years it was ≥ 95% ( http://http:/apps.who.int/gho/data/node.main.A827?lang=en). Despite the fact that > 95% children in Poland have received routine primary vaccination with three doses of DTP and one booster within the first 2 years of life only 46.5% of them demonstrated anti-diphtheria antibodies level ≥ 0.1 IU/ml. The low geometric mean in population of children ≥ 2 years old might be related to the lack of adherence to the vaccination schedule. We have not verified this hypothesis as we have no data concerning the vaccination calendar for each tested individual. Nevertheless, this hypothesis may be unlikely because CDC states that interruption of the recommended schedule or delay of subsequent doses does not reduce the response to the vaccine when the series is finally completed [13].

The antibody level in the Polish population increased with each booster dose and achieved the highest level in young adults (19–25 years) after the last booster administered at age 19 years. It is worth underlining that overall the proportion of persons with a high level of anti-diphteria toxoid IgG antibodies (>1.0 IU/ml), which confers long-term protection, was only 6.1%. These results differ significantly from those obtained in a previous study concerning the prevalence of diphtheria immunity in the Polish population conducted in 1990s by Walory et al. [3]. These authors showed that 23% individuals examined were seronegative, 64% showed basic protection (0.1-1.0 IU/ml) and 13% were highly protected (>1.0 IU/ml). The higher level of diphtheria antitoxin antibodies in the Polish population in the 1990s may have been maintained by reinforcing natural immunity and boosting through subclinical infections, as in 1990s and earlier, diphtheria was a quite common disease in Poland. Such an explanation is supported by the observation in Latvia where diphtheria is endemic and the prevalence of antibodies to diphtheria toxin is high in all age groups of the population (investigated using a commercial ELISA kit) [14].

Ohuabunwo et al. [15] revealed that individuals who received a booster dose of an anti-diphtheria vaccine with higher-antigen concentration DT as oppose to the lower-antigen concentration dT had higher diphtheria toxin antibody levels. According to Polish recommendations children up to 6 years old received DTP or DT vaccine containing ≥30 IU of diphtheria toxoid, whereas teenagers and adults received dT vaccine containing ≥ 0.5 IU of diphtheria toxoid. Administration of the higher-antigen concentration anti-diphtheria vaccine to children did not give the expected higher anti-diphtheria antibodies levels. There remains a question as to whether administration of DT to teenagers and adults instead of dT would cause longer persistence of high anti-diphtheria antibodies levels in these persons. This problem should be investigated in the near future.

Studies concerning seroepidemiology of diphtheria in Western Europe revealed that the proportion with serum antibodies to diphtheria toxin rises with age in children, which is in agreement with our findings [16]. But in our studies <50% children in age 0–2 years old had protective level of antibodies whereas Edmunds et al. [16] showed that >90% of 1 year olds were seropositive (various tests were used, such as ELISA, DELFIA, ToBI and the Vero cell neutralization test). A low proportion of seropositive children was also identified in the Czech Republic (using the Vero cell neutralization test) [14]. Our results together with results obtained for Czech population support the opinion of Chironna et al. [17], that basic immunization without booster doses may result in unsatisfactory protection among children. The differences in the proportion of seropositive children may be a result of the application of different vaccines. Poland is the only European country which uses the DTP vaccine containing a whole-cell pertussis component (DTwP). The acellular vaccine (DTaP) is also available, but is not refunded by the government therefore the overwhelming majority of children are vaccinated using the DTwP. It is documented in the scientific literature that antibody responses to all antigens in DTwP vaccines can be lower compared to DTaP vaccines [18,19]. However, some authors showed that antibody response to the diphtheria toxoid antigen is similar in DTwP and DTaP vaccines [20-23]. Considering the above discrepancies as well as the results of our study, it would be prudent to reevaluate the immunogenicity of the DTwP vaccine used in Poland in comparison to DTaP vaccines registered in Poland. Results of this evaluation may lead to changes in recommendations for immunization which could improve diphtheria immunity in the Polish population and reduce the risk of diphtheria infection.

In all European countries where diphtheria seroprevalence studies were conducted, including Poland, the level of antibodies to diphtheria toxin decreases significantly in persons above 40 years old [14,16,24-29]. The most drastic decrease was observed in Poland (this study), Spain and Ireland, reaching > 67% of seronegative individuals [14,25].

No significant sex-related differences in the proportion of seropositive individuals were identified in Poland, although, in some countries marked sex-related differences were observed with higher number of seropositive males. This is probably due to the fact that in those countries military recruits had been vaccinated against diphtheria [26-29].

Measuring the amount of serum antibodies against diphtheria toxin in individuals is the only way to survey the level of protection in a community. Even though the use of different methods and test kits for determination of the anti-diphtheria antibodies level may influence the results obtained [11], the comparison of seroprevalence in various European countries clearly demonstrate that a high percentage of adults is not protected against diphtheria. In Poland, similar to several other European countries, booster doses every 10 years in adults are recommended. However, it is difficult to monitor vaccination coverage in adults and, as suggested by our results, diphtheria booster vaccination in adults is uncommon. A national information campaign should be organized to inform the public about the importance of following vaccination recommendations and to persuade the public that diphtheria vaccines are useful not only for children but also for adults. A special effort should be made to remind health-care workers, especially general practitioners, about the importance of diphtheria booster vaccinations every 10 years. Adaptation of vaccination strategies should also be considered such that every 10 year a general recommendation would be issued (e.g., at age 40, 50, 60, etc.). This would help patients and doctors remember and monitor booster doses.

Despite the high proportion of seronegative individuals in Polish population, diphtheria cases have not been recorded for over 10 years. It could be argued that toxigenic corynebacteria do not circulate in the population. But it must be kept in mind that the disease could be imported from endemic regions and cause an outbreak in a susceptible community.

## Conclusions

This study showed that there is insufficient herd immunity to diphtheria in the Polish population which could potential lead to an epidemic. Before the introduction of diphtheria vaccination, diphtheria was predominantly known as a childhood infection. Currently the disease affects mainly adults [1,8,9]. Due to the low levels of anti-diphtheria antibodies in persons > 40 years old, the development of tourism to diphtheria endemic regions and the newly identified source of infection, i.e., domestic cats and dogs, it seems reasonable to carry out a publicity campaign regarding recommendations for diphtheria booster vaccination in adults. Special attention should be paid to travelers and persons taking care of cats and dogs. Moreover, due to the low proportion of seropositives found in children ≤ 2 years old the immunogenicity of the DTP vaccine used in Poland should be verified. Full protection in the highest possible proportion of the population should help to avoid re-emergence of this serious, potentially fatal infectious disease.

## Competing interests

The authors declare that they have no competing interests.

## Authors’ contributions

AAZ designed the study, analyzed the results, reviewed the literature and prepared the manuscript; WR selected the serum samples, conducted statistical analyses, participated in analysis of results and the manuscript preparation; NR conducted serological tests; MJ contributed to drafting the manuscript. All the authors read and approved the final manuscript.

## Pre-publication history

The pre-publication history for this paper can be accessed here:

http://www.biomedcentral.com/1471-2334/13/551/prepub
